# ACE2 Correlated With Immune Infiltration Serves As A Novel Prognostic Biomarker In Clear Cell Renal Cell Carcinoma: Implication For COVID-19

**DOI:** 10.7150/ijbs.51969

**Published:** 2021-01-01

**Authors:** Wuping Yang, Lei Li, Kenan Zhang, Kaifang Ma, Haibiao Xie, Yanqing Gong, Jingcheng Zhou, Kan Gong

**Affiliations:** 1 Department of Urology, Peking University First Hospital, Beijing 100034, P.R. China; 2 Hereditary Kidney Cancer Research Center, Peking University First Hospital, Beijing 100034, P.R. China; 3 National Urological Cancer Center, Beijing 100034, P.R. China

**Keywords:** COVID-19, ACE2, ccRCC, prognosis, immune infiltration

## Abstract

The current severe acute respiratory syndrome coronavirus 2 (SARS-CoV-2) has caused a global infection, and is seriously threatening human life, especially cancer patients. Thus, we sought to determine the clinical roles of ACE2 (the cell entry receptor of SARS-CoV-2) in ccRCC (clear cell renal cell carcinoma). TCGA, GEO and TIP datasets, and immunohistochemistry and western blot results were used to determine the prognostic and clinicopathological characteristics of ACE2. ACE2 expression was down-regulated in ccRCC tissues and cell lines. The multivariate Cox regression analysis results indicated that increased ACE2 expression was independent predictor of longer OS (HR: 0.8259, 95%CI: 0.7734-0.8819, P<0.0001) and RFS (HR: 0.8023, 95%CI: 0.7375-0.8729, P<0.0001) in ccRCC patients. Lower ACE2 expression was also associated with advanced tumor stage, higher histological grade and pathological stage, and metastasis. Besides, ACE2 expression was significantly positively and negatively correlated with CD4 Naïve infiltration and CD4 Memory infiltration, respectively. Moreover, higher CD4 Naïve and lower CD4 Memory infiltration levels were associated with better pathological features and longer OS and RFS. Furthermore, high ACE2 expression group in decreased CD4 Naïve, enriched CD4 Naïve and enriched CD4 memory cohort had favorable prognosis. These findings identified that AEC2 was significantly reduced in ccRCC, and decreased ACE2 was related to worse pathological features and poor prognosis. Low ACE2 expression in ccRCC may partially affect the prognosis due to altered immune cells infiltration levels.

## Introduction

Coronavirus disease 2019 (COVID-19) is an infectious disease caused by severe acute respiratory syndrome coronavirus 2 (SARS-CoV-2, formerly known as 2019-nCoV) 1, 2. At the end of 2019, COVID-19 has been detected in Wuhan City, Hubei Province, China 2. As of July 3, 2020, a total of 10662536 confirmed cases and 516209 deaths caused by COVID-19 have been reported globally according to World Health Organization (https://covid19.who.int/).

Angiotensin-converting enzyme 2 (ACE2) is known as a host cell receptor for severe acute respiratory syndrome coronavirus (SARS-CoV) 3. It has been confirmed that SARS-CoV-2 and SARS-CoV have 79.5% homologous sequences, and it also utilizes ACE2 as cellular entry receptor 4. The structural Spike (S) protein binds to the cellular receptor ACE2 to drive the entry of the SARS-CoV and SARS-CoV-2 into target host cells. Similar to the SARS-CoV infection, the interaction between the S protein of SARS-CoV-2 and the cellular ACE2 helps the virus attach to the target cells 5. Previous in vitro studies have also shown that elevated ACE2 expression promotes susceptibility to the S protein-driven infections 6, 7. Thus, in healthy individuals, organs such as lung, heart, kidney, bladder and esophagus that express high levels of ACE2 seem to be more susceptible to SARS-CoV-2 infection 8-10.

SARS-CoV-2 infection usually manifests as fever and dry cough, but there are other clinical manifestations, including fatigue, dyspnea, nasal congestion, nausea, or diarrhea 11, 12. Besides, those with pre-existing conditions, such as diabetes, hypertension, and pulmonary, cardiac, and kidney diseases are thought to have a higher risk of developing serious disease 13-15. At present, there are more than 18 million new cancer cases in the world every year 16, and cancer patients are more likely to be infected because of poor health status, accompanying chronic diseases, and immunosuppressive conditions caused by tumors and anti-tumor treatments 17. Liang et al. reported that among 1590 SARS-CoV-2 subjects, 18 (1%) had a history of tumors, which was higher than the occurrence of tumors in the general Chinese population (0.29%) 18. Miyashita et al evaluated the clinical and demographic information of 5688 patients with SARS-CoV-2, and found 334 subjects (6%) with tumors in this group 19. A report from Italy indicated that 20% of subjects with SARS-CoV-2 had been treated for a tumor in the previous 5 years 20. Therefore, tumor patients with SARS-CoV-2 coronavirus infection may have a worse outcome than other subjects.

In this study, we demonstrated the expression profile of ACE2 in ccRCC (clear cell renal cell carcinoma) and adjacent normal tissues, and determined the prognostic roles of ACE2 in ccRCC and its relationship with clinicopathological characteristics and immune infiltration based on TCGA, GEO and TIP databases. Besides, we confirmed the expression of ACE2 protein in 56 paired ccRCC tissues (ccRCC and adjacent normal tissue), and validated the prognostic and clinicopathological roles of ACE2 in ccRCC. Furthermore, we detected the expression of three immune cell markers (CD4, CD45RA and CD45RO) and analyzed their correlation with ACE2.

## Materials and Methods

### Ethics statement

This study was approved by the Biomedical Research Ethics Committee of Peking University First Hospital (Beijing, China, IRB00001052-18004). Written informed consents were also obtained from all patients.

### TCGA, GEO and TIP databases

RNA-sequencing and clinical follow-up (overall survival (OS) and relapse-free survival (RFS)) data of 530 ccRCC tissues were downloaded from TCGA. RNA-sequencing data of GSE40435, GSE53757, GSE66272, GSE126964 and GSE73731 datasets was obtained from GEO. The data of immune cells infiltration in TCGA-KIRC was gained from TIP -- Tracking Tumor Immunophenotype.

### Immunohistochemical examination

54 pairs of matched paraffin section samples (ccRCC and adjacent normal tissue) were utilized to perform immunohistochemistry staining to measure the protein expression of ACE2, CD45RA and CD45RO. The specific primary antibody information is as follows: anti-ACE2 (Affinity, AF5165, 1:100 dilution), anti-CD45RA (Abcam, ab755, 1:2000 dilution) and anti-CD45RO (Abcam, ab23, 1:1000 dilution).

### Western blot analysis

The normal human renal tubular epithelial cell line HK2 and several ccRCC cell lines including 786-O, OSRC2 and A498 were used in this study. Protein was extracted from these cell lines using the RIPA buffer, and BCA method was utilized to quantitate the total protein levels. Then protein was separated by SDS-PAGE and transblotted to PVDF membranes. The membranes were blocked using 5% skimmed milk powder and incubated overnight at 4 °C with anti-ACE2 (Affinity, AF5165, 1:1000 dilution) and anti-GAPDH (Proteintech, Lot:00075847, 1:10000 dilution).

### Statistical analysis

Non-parametric Mann-Whitney test was used to detect differences in continuous variables. The Pearson's correlation test was performed to examine the correlation between ACE2 expression and the infiltration of immune cells and the expression of immune cell markers. The prognostic value of ACE2 was examined by Kaplan-Meier curve, and log-rank test was utilized to demonstrate the significance of the difference in survival curves. The univariate and multivariate Cox regression analyses of ACE2 were conducted. A P-value < 0.05 indicated statistical significance.

## Results

### ACE2 expression was down-regulated in ccRCC tissues and cell lines

**Figure 1:**
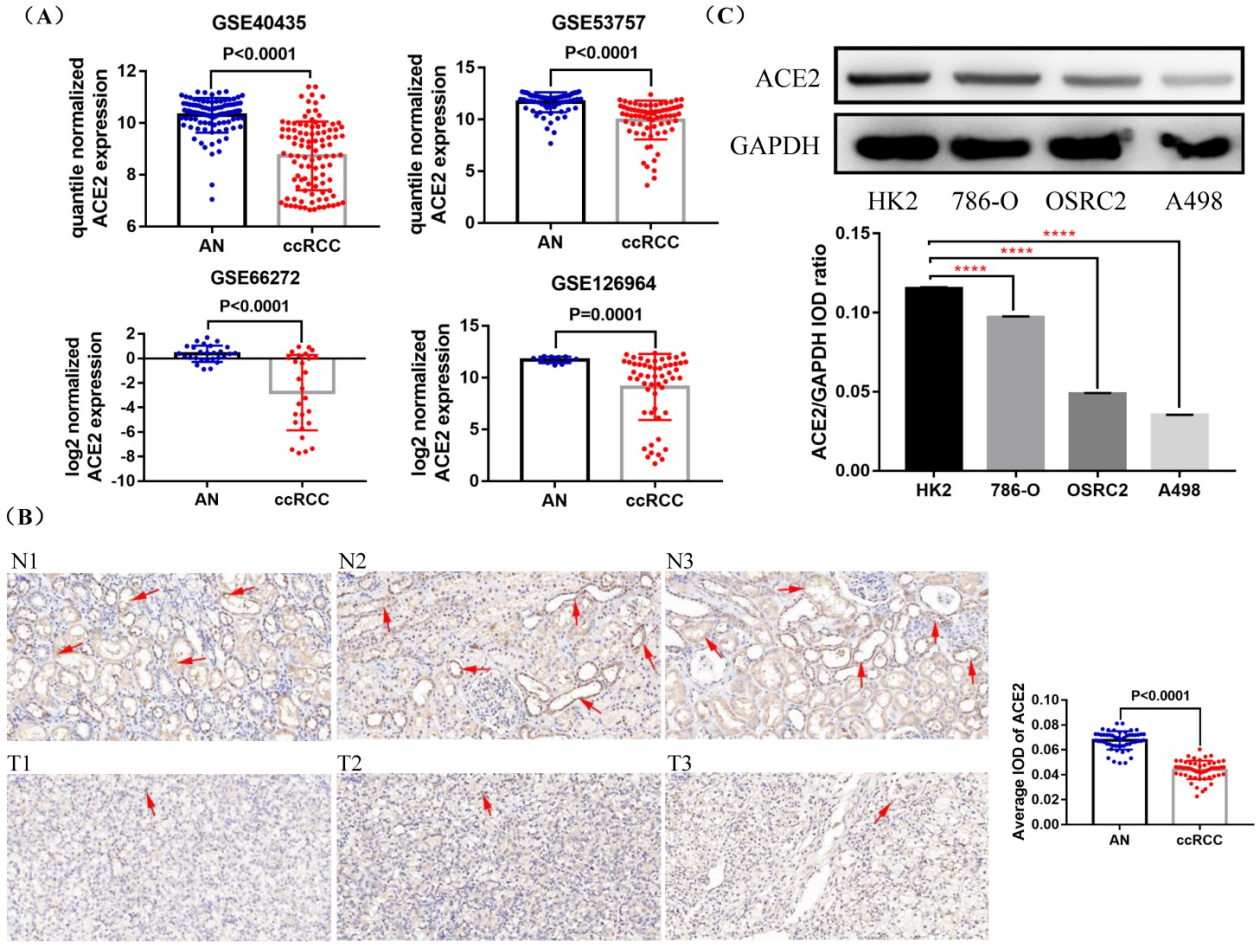
Expression status of ACE2 in ccRCC tissues and cell lines. (A) Comparison of ACE2 mRNA expression in ccRCC and adjacent normal tissues (AN) based on GSE40435, GSE53757, GSE66272 and GSE126964 datasets. (B) Comparison of ACE2 protein expression in 54 pairs of matched paraffin section samples (ccRCC and adjacent normal tissue) using immunohistochemistry (C) Comparison of ACE2 protein expression in HK2 cell line and three ccRCC cell lines (786-O, OSRC2 and A498). ****p < 0.0001

First, we compared the expression of ACE2 mRNA in ccRCC and adjacent normal tissues based on four GEO datasets (GSE40435, GSE53757, GSE66272 and GSE126964), and found that ACE2 mRNA expression was down-regulated in ccRCC tissues compared with adjacent normal tissues (Figure 1A). To further determine the expression pattern of ACE2 protein in ccRCC, we examined ACE2 protein expression in 54 ccRCC tissues and their corresponding adjacent normal tissues, and the detailed clinical features of the 54 patients were provided in Table 1. Consistent with the results of GEO data analysis, immunohistochemistry results showed that ACE2 protein expression was significantly lower in ccRCC tissues than that in adjacent normal tissues (Figure 1B). Besides, the western blot results confirmed the decreased expression of ACE2 protein in ccRCC cell lines (Figure 1C).

### Decreased ACE2 expression was associated with worse prognosis

**Figure 2:**
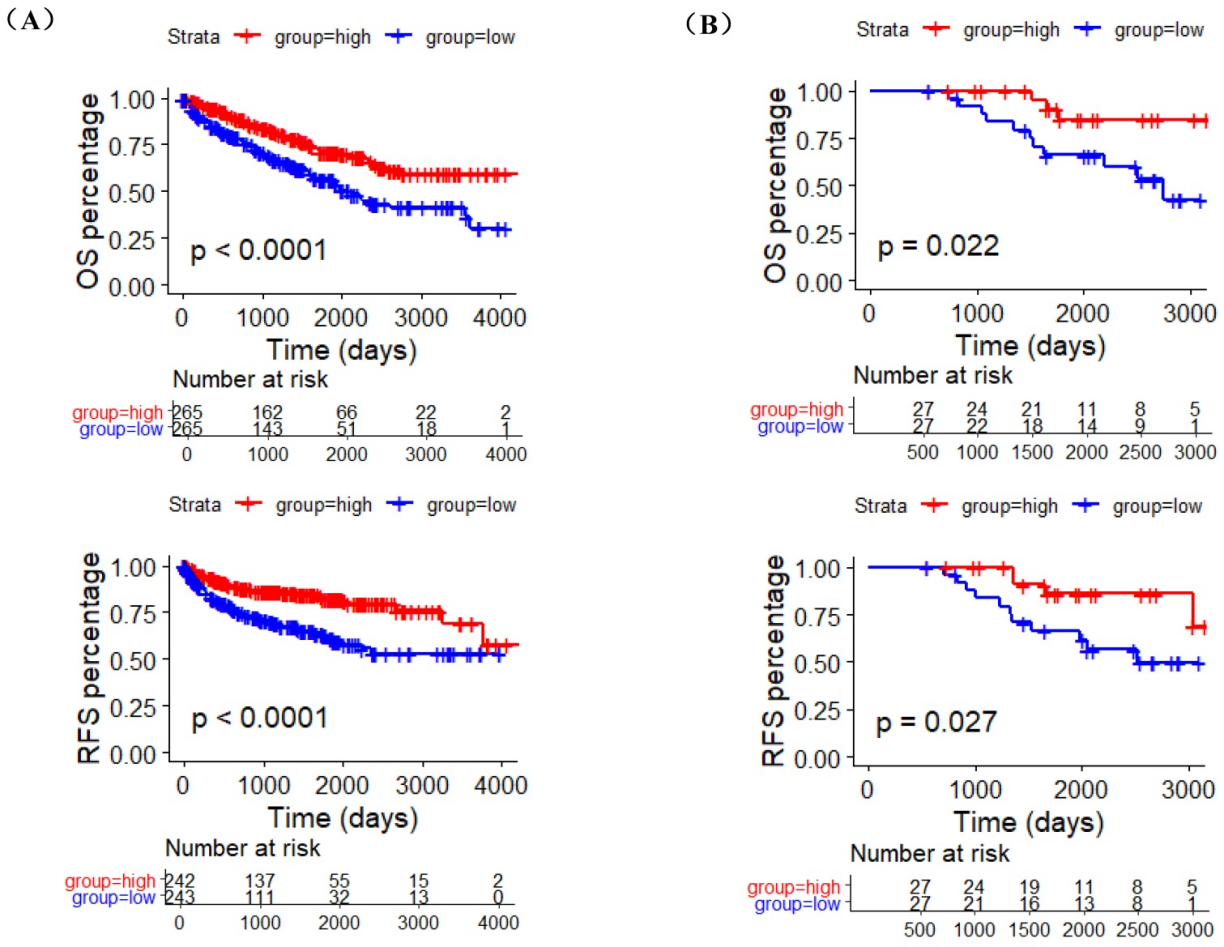
The prognostic value of ACE2 in ccRCC patients. (A) K-M curves of OS and RFS in 530 and 485 TCGA ccRCC patients, respectively. (B) K-M curves of OS and RFS in 54 clinical ccRCC patients. Patients were separated into two groups according to the median cutoff of ACE2 expression.

In order to determine the prognostic value of ACE2 in ccRCC, we obtained the RNA-sequencing and clinical follow-up data of 530 ccRCC samples from TCGA. The 530 patients were divided into two groups according to the median critical value of CLDN10 expression, and log-rank test results showed that the high ACE2 expression group had remarkably longer OS and RFS than the low ACE2 expression group (Figure 2A). Moreover, univariate Cox regression analysis results showed that advanced tumor stage (T3/T4), metastasis (M1), higher pathological stage (Stage III/IV) and histological grade (Grade 3/4) were potential risk factors of poor OS, while increased ACE2 expression was related to better OS. Then, the multivariate Cox regression analysis results determined that M1, Stage III/IV and Grade 3/4 were independently associated with shorter OS, while increased ACE2 expression independently predicted longer OS (HR: 0.8259, 95%CI: 0.7734-0.8819, P<0.0001) (Table 2). In terms of RFS, T3/T4, M1, Stage III/IV and Grade 3/4 were potential risk factors of poor RFS, while increased ACE2 expression was associated with better RFS. The following multivariate Cox regression analysis results demonstrated that M1, Stage III/IV and Grade 3/4 were independent predictors of shorter DFS, while increased ACE2 expression (HR: 0.8023, 95%CI: 0.7375-0.8729, P<0.0001) was independent predictor of longer RFS in ccRCC patients (Table 3). Furthermore, we verified the prognostic roles of ACE2 in ccRCC by combining the ACE2 protein expression data of 54 clinical ccRCC samples and their OS and RFS information (Figure 2B).

### Lower ACE2 expression was associated with worse pathological features

**Figure 3:**
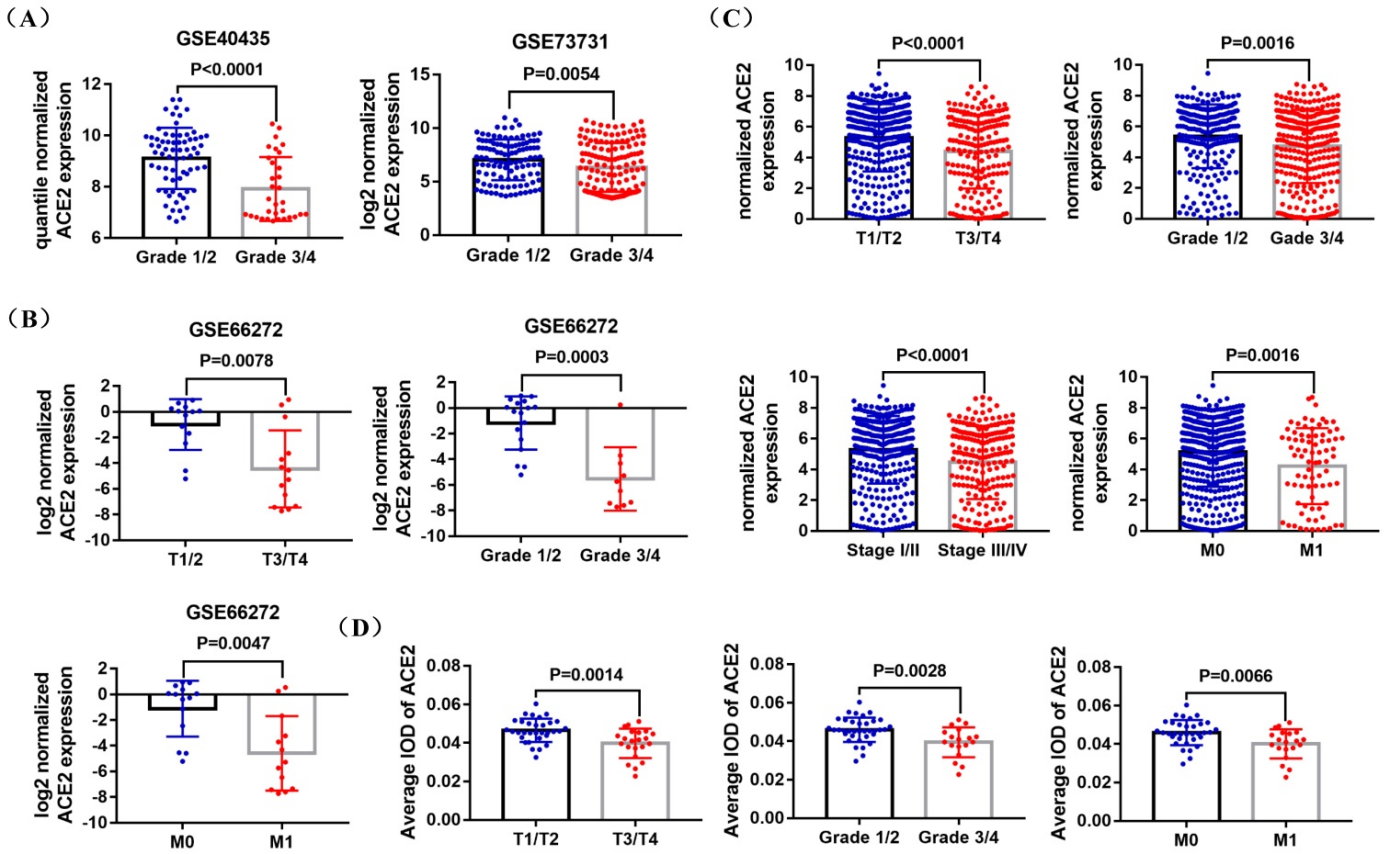
Correlation between ACE2 expression and the pathological features of ccRCC patients. (A) Comparison of ACE2 mRNA expression in Grade 1/2 and Grade 3/4 patients based on GSE40435 and GSE73731 datasets. (B) Comparison of ACE2 mRNA expression in T1/T2 and T3/T4 patients, Grade 1/2 and Grade 3/4 patients, and M0 and M1 patients based on GSE66272 dataset. (C) Comparison of ACE2 mRNA expression in T1/T2 and T3/T4 patients, Grade 1/2 and Grade 3/4 patients, Stage I/II and Stage III/IV patients, and M0 and M1 patients based on TCGAKIRC data. (D) Comparison of ACE2 mRNA expression in T1/T2 and T3/T4 patients, Grade 1/2 and Grade 3/4 patients, and M0 and M1 patients based on the immunohistochemistry results of 54 ccRCC paraffin section samples.

**Figure 4:**
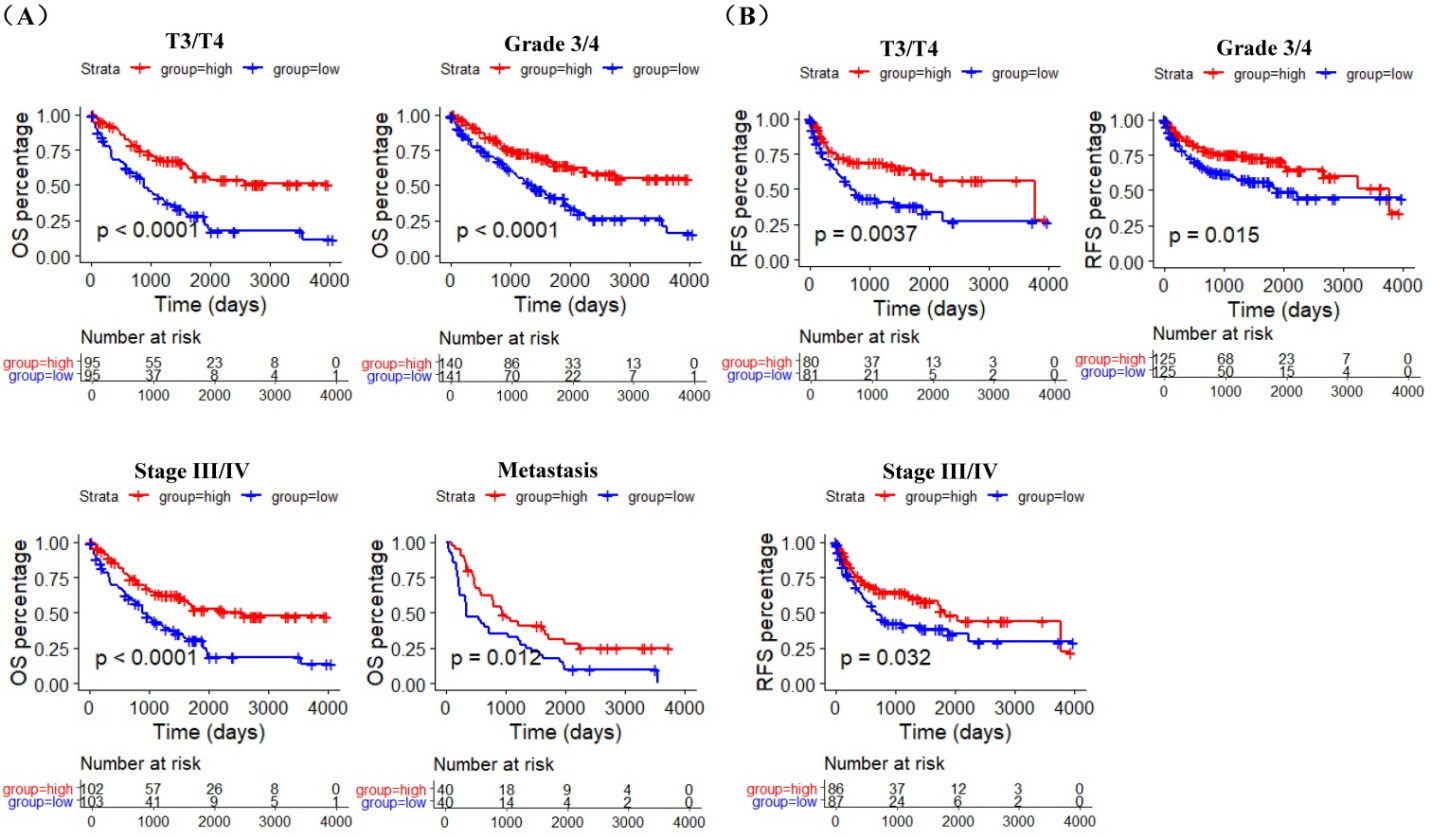
The prognostic value of ACE2 in T3/T4, Grade 3/4, Stage III/IV and Metastasis subgroups. (A) K-M curves of OS in TCGA T3/T4, Grade 3/4, Stage III/IV and metastasis ccRCC patients respectively. (B) K-M curves of RFS in TCGA T3/T4, Grade 3/4 and Stage III/IV ccRCC patients respectively. Patients were separated into two groups according to the median cutoff of ACE2 expression.

GSE40435, GSE73731, GSE66272 datasets and TCGA RNA-sequencing and clinical data was used to explore the correlation between ACE2 expression and pathological features of ccRCC patients. The analysis results of GSE40435 and GSE73731 datasets showed that Grade 3/4 groups had lower ACE2 expression (Figure 3A), and the analysis results of GSE66272 dataset indicated that T3/T4, Grade 3/4 and M1 groups also had lower ACE2 expression (Figure 3B). Besides, the analysis results of TCGA data demonstrated that lower ACE2 expression was associated with advanced tumor stage, higher histological grade and pathological stage, and metastasis (Figure 3C). In addition, our immunohistochemistry results confirmed the lower ACE2 expression in T3/T4, Grade 3/4 and M1 groups (Figure 3D). Furthermore, we used the Kaplan Meier curve to check the prognostic value of ACE2 in different subgroups (T3/T4, Grade 3/4, Stage III/IV and Metastasis subgroups). The log-rank test results showed that high ACE2 expression groups was associated with longer OS in T3/T4, Grade 3/4, Stage III/IV and Metastasis subgroups (Figure 4A), and it was also correlated with longer RFS in T3/T4, Grade 3/4 and Stage III/IV subgroups (Figure 4B).

### Prognostic and clinicopathological roles of immune cells in ccRCC

**Figure 5:**
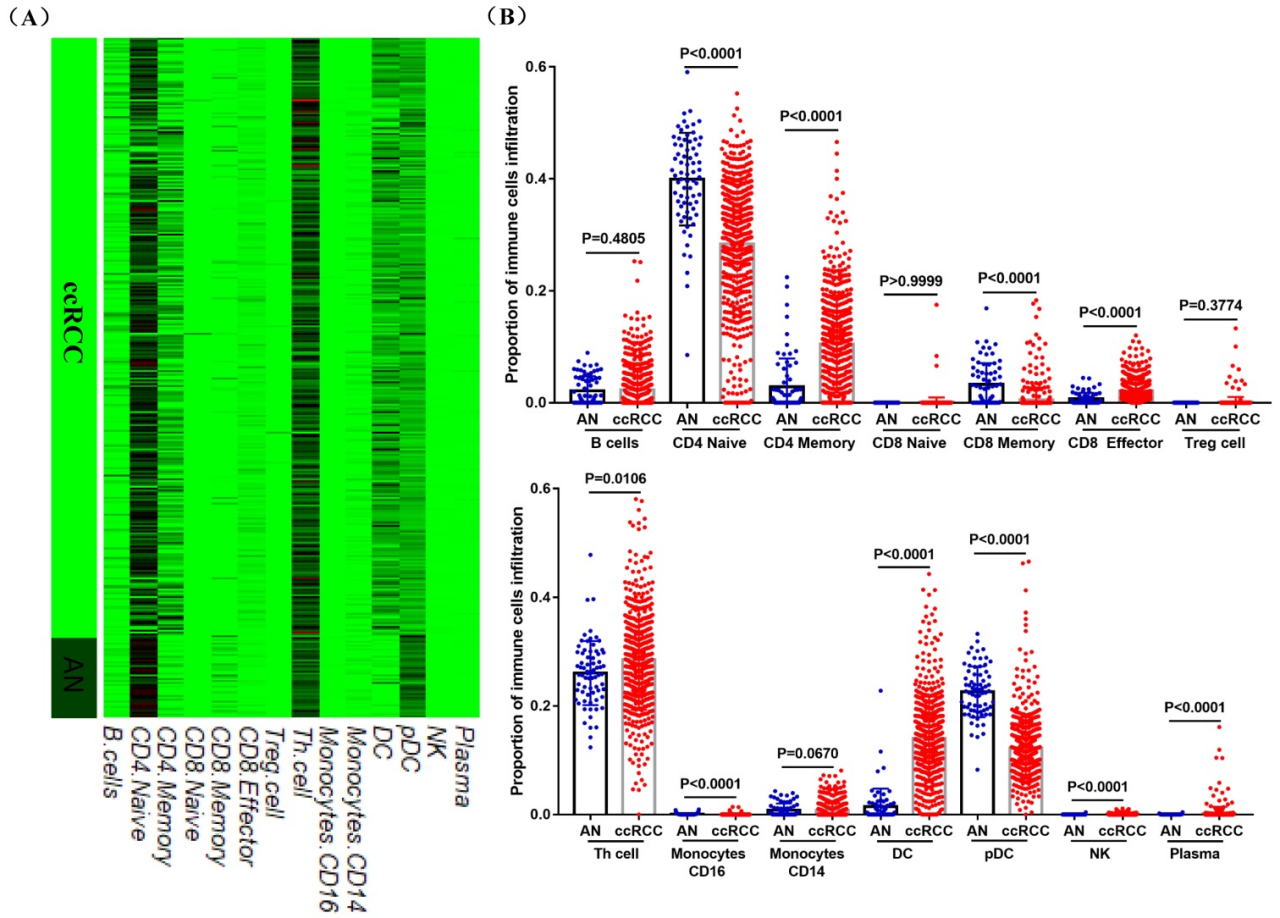
The infiltration levels of 14 kinds of immune cells in ccRCC and adjacent normal tissues. Heatmap (A) and statistical comparison (B) of the difference in the infiltration levels of 14 kinds of immune cells (B cells, CD4 Naïve, CD4 Memory, CD8 Naïve, CD8 Memory, CD8 Effector, Treg cell, Th cell, Monocytes CD16, Monocytes CD14, DC, pDC, NK and Plasma) in ccRCC and adjacent normal tissues. All the data were from TIP.

ccRCC has been proved to be a highly immune infiltrating tumor based on various studies 21, 22, and tumor infiltration was related to the prognosis of RCC patients 23, 24. However, the specific effect of immune cells on the prognosis of ccRCC patients and its relationship with the pathological characteristics of ccRCC are still vague. Using data from TIP and TCGA, we compared the infiltration levels of 14 kinds of immune cells (B cells, CD4 Naïve, CD4 Memory, CD8 Naïve, CD8 Memory, CD8 Effector, Treg cell, Th cell, Monocytes CD16, Monocytes CD14, DC, pDC, NK and Plasma) in ccRCC and adjacent normal tissues, and their correlation with the prognostic and clinicopathological roles of ccRCC patients were also tested. Results showed that the infiltration levels of CD4 Naive, CD8 Memory, Monocytes CD16 and pDC were decreased in ccRCC compared with adjacent normal tissues, and the infiltration levels of CD4 Memory, CD8 Effector, Th cell, DC, NK and Plasma were increased in ccRCC than that in adjacent normal tissues (Figure 5A and 5B).

**Figure 6:**
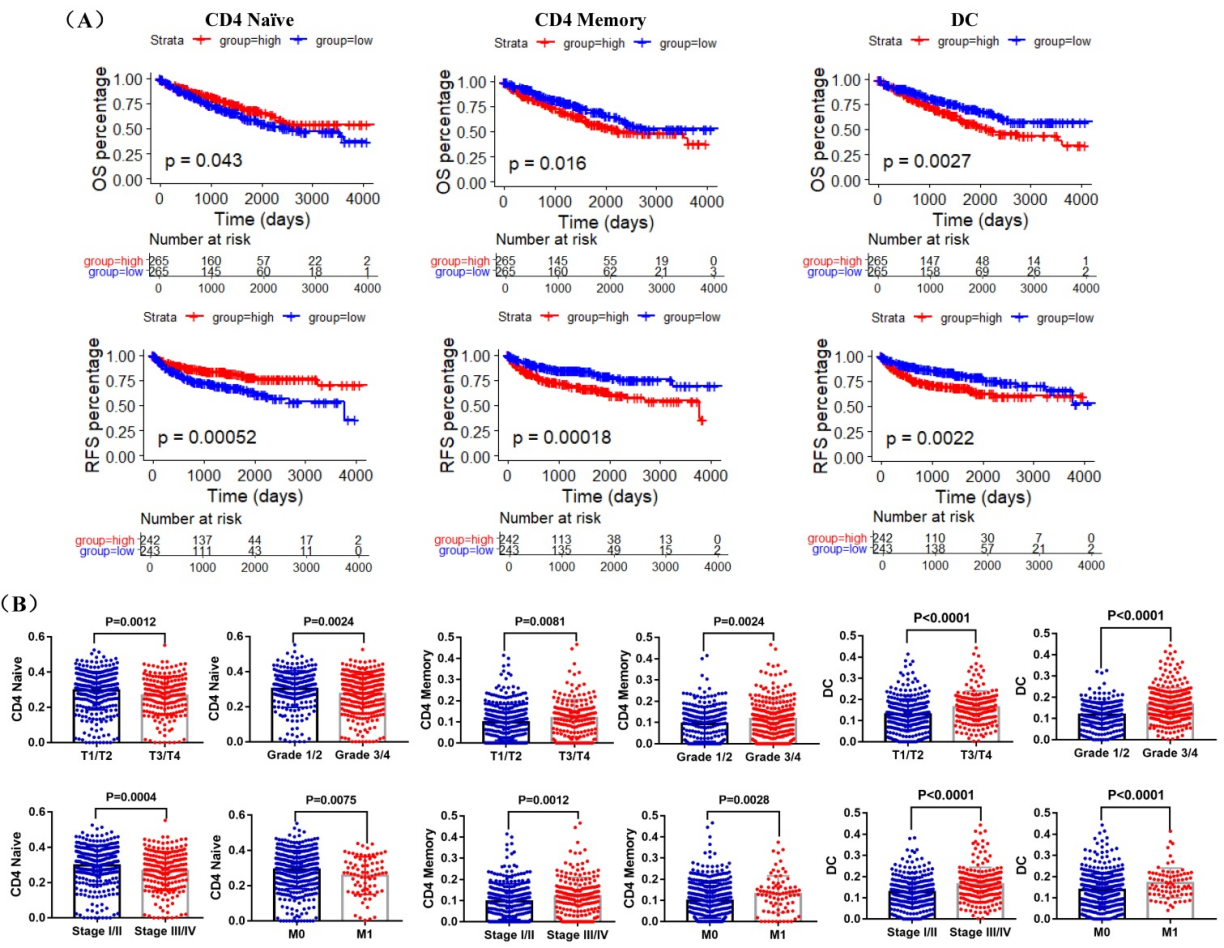
Prognostic and clinicopathological roles of immune cells in ccRCC. (A) K-M curves of OS and RFS in TCGA ccRCC patients. Patients were separated into two groups according to the median cutoff of ACE2 CD4 Naïve, CD4 memory and DC infiltration levels. (B) Comparison of CD4 Naïve, CD4 memory and DC infiltration levels in T1/T2 and T3/T4 patients, Grade 1/2 and Grade 3/4 patients, Stage I/II and Stage III/IV patients, and M0 and M1 patients based on TCGA data.

Kaplan-Meier curve was used to examine the prognostic roles of these differentially distributed immune cells in ccRCC, and log-rank test results indicated that only the infiltration levels of CD4 Naïve, CD4 Memory and DC cells were related to both OS and RFS. Higher CD4 Naïve infiltration level was associated with longer OS and RFS, while lower infiltration levels of CD4 Memory and DC were connected with longer OS and RFS (Figure 6A). However, there was no difference in OS and RFS between the high and low infiltration level groups of CD8 Effector, Th cell, Monocytes CD16 and NK, and the infiltration levels of CD8 Memory, pDC and Plasma were only related to RFS (Supplementary Figure 1). Moreover, T3/T4, G3/G4, Stage III/IV, and Metastasis (M1) groups had lower CD4 Naïve and higher CD4 Memory and DC infiltration levels (Figure 6B).

### ACE2 expression was associated with CD4 Naïve and CD4 Memory infiltration

**Figure 7:**
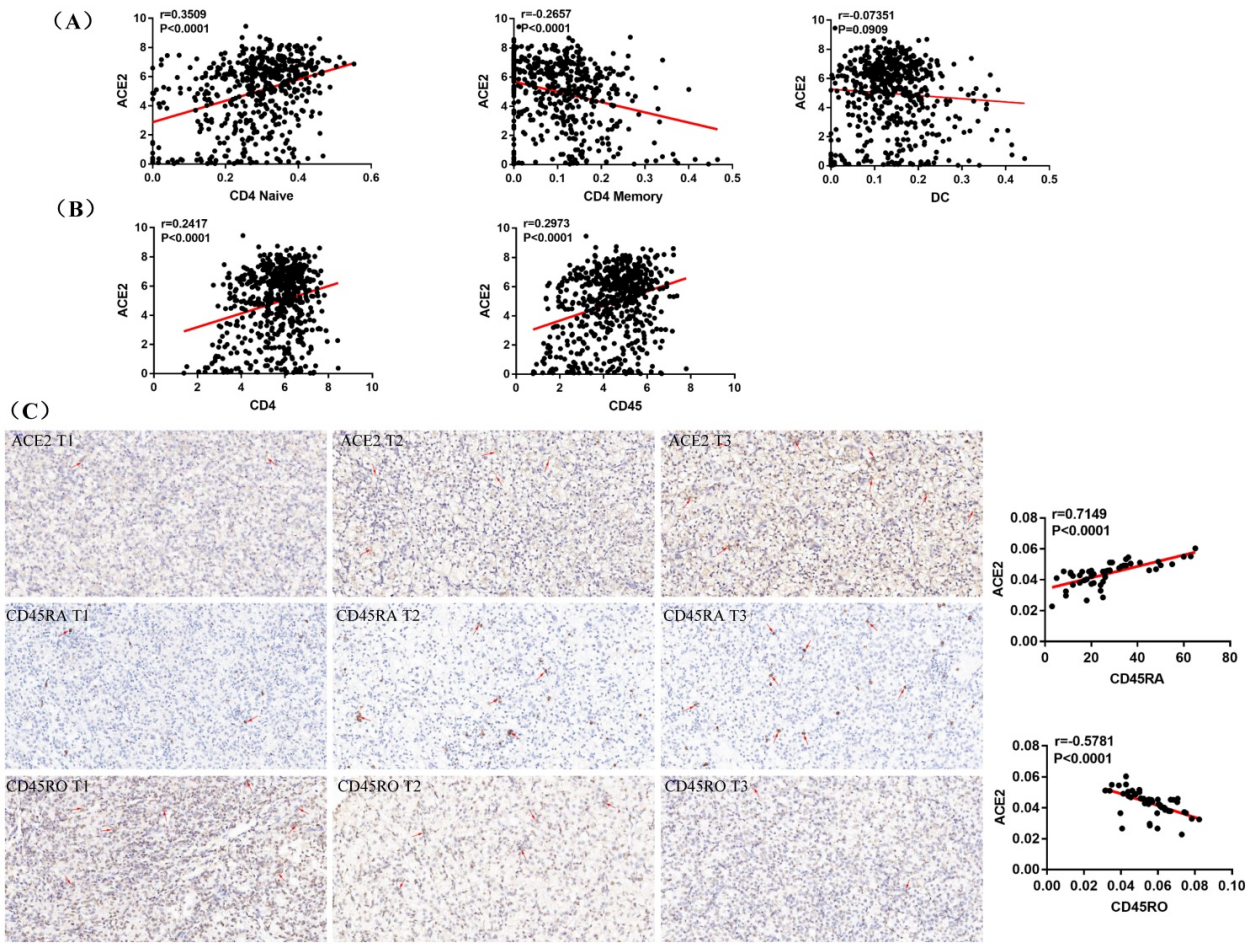
ACE2 expression was associated with CD4 Naïve and CD4 Memory infiltration. (A) Correlation between ACE2 expression and CD4 Naïve, CD4 Memory and DC infiltration levels based TCGA and TIP data. (B) Correlation between ACE2 expression and CD4 and CD45 expression based on TCGA data. (C) Correlation between ACE2 protein expression and CD45RA and CD45RO protein expression based on the immunohistochemistry staining results of 54 clinical ccRCC samples.

**Figure 8:**
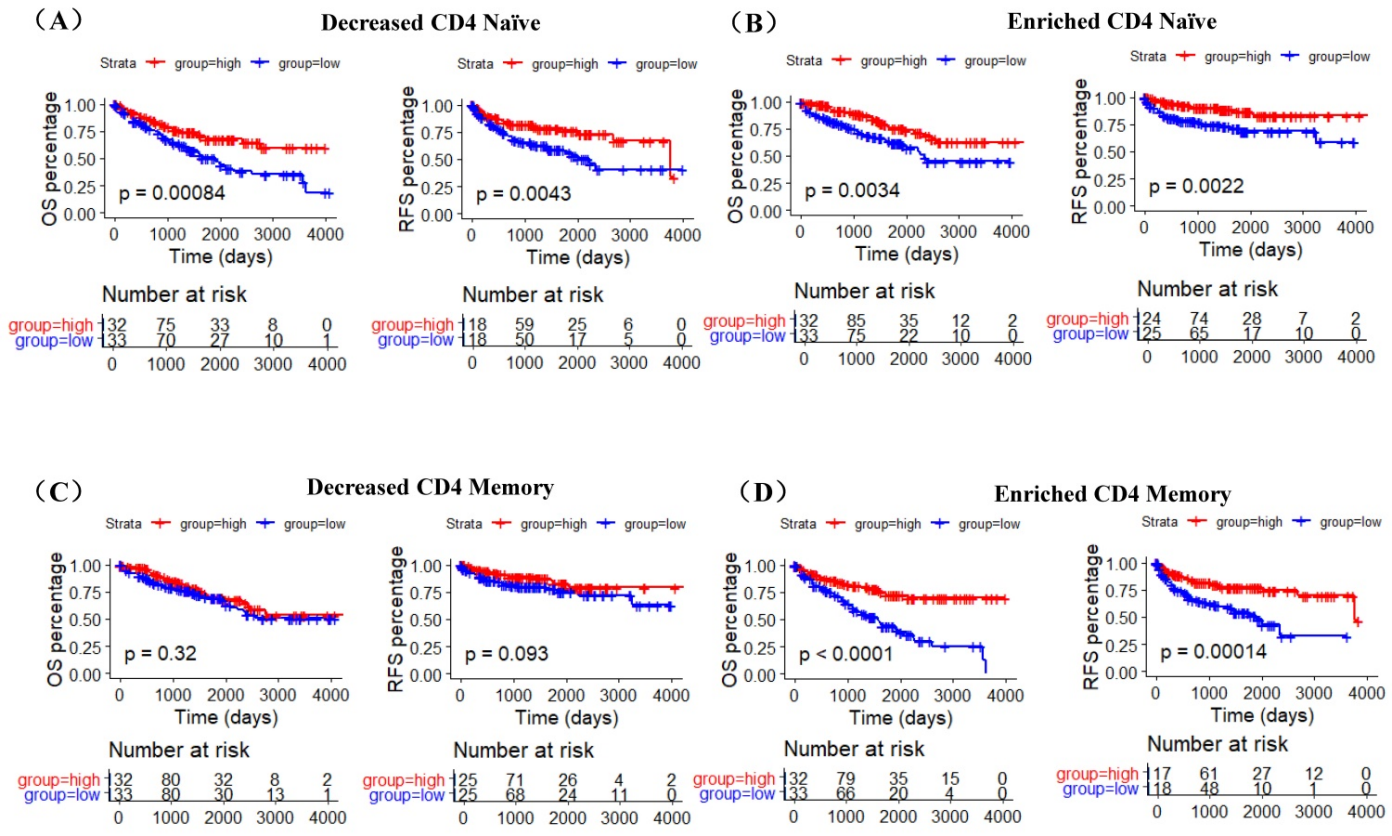
The prognostic roles of ACE2 in relevant immune cell subgroups. K-M curves of OS and RFS in decreased CD4 Naïve (A), enriched CD4 Naïve (B), decreased CD4 memory (C) and enriched CD4 memory (D) cohort. Patients were separated into two groups according to the median cutoff of ACE2 expression.

A recent study indicates that ACE2 expression is correlated with immune infiltration and serves as a prognostic biomarker in multiple tumors 25. To explore whether ACE2 is related to immune cells infiltration in ccRCC, linear regression analysis was utilized to examine the correlation between ACE2 expression and the CD4 Naïve, CD4 Memory and DC infiltration levels. Results showed that ACE2 expression was significantly positively correlated with CD4 Naïve infiltration and negatively correlated with CD4 Memory infiltration, but not significantly correlated with DC infiltration (Figure 7A). Since CD4 and CD45RA were used to screen CD4 Naïve cells, and CD4 and CD45RO were used to identify CD4 Memory cells, we also examined the correlation between ACE2 expression and CD4 and CD45 expression. Results showed that ACE2 expression was remarkably positively correlated with CD4 and CD45 expression (Figure 7B). In addition, we used immunohistochemistry to examine the correlation between ACE2 protein levels and CD45RA and CD45RO protein levels in 54 ccRCC samples, and our results determined that ACE2 expression was positively correlated with CD45RA expression and negatively correlated with CD45RO expression (Figure 7C). Furthermore, we analyzed the prognosis according to the expression level of ACE2 in relevant immune cell subgroups. The results showed that high ACE2 expression group in decreased CD4 Naïve, enriched CD4 Naïve and enriched CD4 memory cohort had longer OS and RFS (Figure 8A-D).

## Discussion

At present, COVID-19 is seriously threatening the safety of human beings all over the world. Cancer patients are more susceptible to infection due to poor health status, accompanying chronic diseases, and immunosuppression caused by tumors and anti-tumor treatments 17. Previous reports also indicate that the prognosis of tumor patients infected with SARS-CoV-2 coronavirus may be worse than that of other patients 18-20. It has been confirmed that SARS-CoV-2 and SARS-CoV share 79.5% homologous sequences and use ACE2 as a cell entry receptor 4. Therefore, determining the expression profile of ACE2 in ccRCC and its relationship with the prognosis of ccRCC patients can help to further understand the impact of COVID-19 on ccRCC patients.

In this study, we found that ACE2 mRNA was down-regulated in ccRCC compared with adjacent normal tissues by analyzing four GEO datasets, and our western blot and immunohistochemistry results also identified that ACE2 protein expression was decreased in ccRCC tissues and cell lines. Using the RNA-sequencing and clinical data from TCGA, survival analysis results showed that the high ACE2 expression group had remarkably longer OS and RFS than the low ACE2 expression group, and the multivariate Cox regression analysis results indicated that increased ACE2 expression independently predicted longer OS and RFS of ccRCC patients. Besides, we verified the prognostic roles of ACE2 in ccRCC by combining the ACE2 protein expression data of 54 clinical ccRCC samples and their OS and RFS information. In addition, lower ACE2 expression was associated with advanced tumor stage, higher pathological stage and histological grade, and metastasis. Moreover, our immunohistochemistry results confirmed that T3/T4, Grade 3/4 and M1 groups had lower ACE2 protein expression than that in T1/T2, Grade 1/2 and M0 groups, respectively. Furthermore, survival analysis results also demonstrated that higher ACE2 expression was associated with better prognosis in T3/T4, Grade 3/4, Stage III/IV and Metastasis subgroups. Taken together, the above analysis and experimental results suggested that ACE2 was low expressed in ccRCC, and higher ACE2 expression was related to better prognosis and pathological features of ccRCC patients.

Several recent studies have shown that tumor-associated immune cells in the tumor associated microenvironment (TAM) play an important role in regulating multiple important biological processes of tumor cells. For example, CD8+ cytotoxic T lymphocytes (CTLs) in TAM have been shown to inhibit tumor progression by targeting tumor-related antigens, and activation of CTLs predicts a better prognosis in various tumors 26. A recent study through the analysis of various databases indicates that ACE2 expression in Uterine Corpus Endometrial Carcinoma and Kidney Renal Papillary Cell Carcinoma was significantly increased, which was positively correlated with immune infiltration and prognosis 25. However, the correlation between ACE2 expression and the infiltration of immune cells in ccRCC remains unclear.

During this study, using data from TIP and TCGA, we compared the infiltration levels of 14 kinds of immune cells (B cells, CD4 Naïve, CD4 Memory, CD8 Naïve, CD8 Memory, CD8 Effector, Treg cell, Th cell, Monocytes CD16, Monocytes CD14, DC, pDC, NK and Plasma) in ccRCC and adjacent normal tissues. Our results indicated that the infiltration levels of CD4 Naive, CD8 Memory, Monocytes CD16 and pDC were decreased in ccRCC compared with adjacent normal tissues, and the infiltration levels of CD4 Memory, CD8 Effector, Th cell, DC, NK and Plasma were increased in ccRCC than that in adjacent normal tissues. Survival analysis results showed that higher CD4 Naïve infiltration level was associated with longer OS and RFS, while lower infiltration levels of CD4 Memory and DC were connected with longer OS and RFS. Besides, lower CD4 Naïve and higher CD4 Memory and DC infiltration levels were associated with advanced tumor stage, higher pathological stage and histological grade, and metastasis. Interestingly, ACE2 expression was significantly positively correlated with CD4 Naïve infiltration and negatively correlated with CD4 Memory infiltration. Moreover, our immunohistochemistry results determined that ACE2 expression was also positively correlated with CD45RA (a CD4 Naïve cell marker) expression and negatively correlated with CD45RO (a CD4 Memory cell marker) expression. Furthermore, we analyzed the prognosis according to the expression level of ACE2 in relevant immune cell subgroups, and results confirmed that high ACE2 expression group in decreased CD4 Naïve, enriched CD4 Naïve and enriched CD4 memory cohort had favorable prognosis. The above results suggested that the low expression of ACE2 in ccRCC may partially affect the prognosis of ccRCC patients due to altered immune cells infiltration levels.

Consistent with our results, previous study also suggested that ACE2 expression was reduced in breast cancer 27, non-small cell lung cancer 28, pancreatic cancer 29 and hepatocellular carcinoma 30, and healthy individuals have increased serum ACE2 activity 31. All these finding demonstrated that lower ACE2 expression levels are frequently associated with the presence of cancer. Besides, recent studies indicated that tumor patients with SARS-CoV-2 coronavirus infection may have a worse outcome than other subjects 18-20. Although ACE2 is the cellular entry receptor of SARS-CoV-2, it is not clear whether ACE2 is involved in the process by which SARS-COV-2 coronavirus infection affects the prognosis of cancer patients. Therefore, the impact of SARS-CoV-2 coronavirus infection on the prognosis of ccRCC patients and the role of ACE2 in this process need to be further studied.

In addition, our study still has some limitations. First, the number of patients enrolled to verify the prognostic value of ACE2 in ccRCC patients needs to be expanded. Second, more in vitro experiments such as immunofluorescence and flow cytometry are needed to screen a kind of specific immune cell to further determine the correlation of ACE2 expression with CD4 naïve T and CD4 memory cells infiltration. Nevertheless, our study is the first to determine the clinical roles of ACE2 in ccRCC through various databases and laboratory experiments, and these findings may contribute to a better understanding of the impact of COVID-19 on ccRCC patients.

## Conclusions

To sum up, our study confirmed that AEC2 was significantly reduced in ccRCC, and decreased ACE2 was associated worse pathological features and poor prognosis. In addition, our study also demonstrated that altered infiltration levels of immune cells may be one of the mechanisms of poor prognosis caused by decreased ACE2 expression.

## Supplementary Material

Supplementary figure.

## Figures and Tables

**Table 1:** The clinicopathological characteristics of 54 ccRCC paraffin section samples

Clinicopathological characteristics		n(%)
Gender		
	Male	32 (59.3)
	Female	22 (40.7)
Tumor size		
	<2cm	4 (7.4)
	>=2cm, <7cm	30 (55.6)
	>=7cm	20 (37.0)
Tumor stage		
	T1/T2	32 (59.3)
	T3/T4	22 (40.7)
Metastasis		
	M0	34 (63.0)
	M1	20 (37.0)
Histological grade		
	Grade 1/2	36 (66.7)
	Grade 3/4	18 (33.3)
Overall survival		
	Alive	34 (63.0)
	Dead	20 (37.0)
Relapse-free survival		
	Non-replased	39 (72.2)
	Replased	15 (27.8)

**Table 2:** Univariate and multivariate Cox regression analysis of OS in 511 TCGA ccRCC patients

	Univariate analysis				Multivariate analysis			
								
Parameters	P	HR	95%CI		P	HR	95%CI	
			Lower	Upper			Lower	Upper
**Tumor stage**								
T1/T2 (63.6%)		1.0000				1.0000		
T3/T4 (36.4%)	**<0.0001**	3.0560	2.2460	4.1570	0.2693	0.7016	0.3741	1.3157
**Metastasis**								
M0 (84.5%)		1.0000				1.0000		
M1 (15.5%)	**<0.0001**	4.3520	3.1860	5.9440	**<0.0001**	2.3027	1.5689	3.3795
**Pathological stage**								
I/II (60.1%)		1.0000				1.0000		
III/IV (39.9%)	**<0.0001**	3.6810	2.6710	5.0740	**0.0061**	2.7272	1.3315	5.5859
**Histological grade**								
Grade 1/2 (45.6%)		1.0000				1.0000		
Grade 3/4 (54.4%)	**<0.0001**	2.6740	1.8910	3.7820	**0.0099**	1.6317	1.1245	2.3676
**ACE2**	**<0.0001**	0.8062	0.7574	0.8580	**<0.0001**	0.8259	0.7734	0.8819

**Table 3:** Univariate and multivariate Cox regression analysis of RFS in 467 TCGA ccRCC patients

	Univariate analysis				Multivariate analysis			
								
Parameters	P	HR	95%CI		P	HR	95%CI	
			Lower	Upper			Lower	Upper
**Tumor stage**								
T1/T2 (66.2%)		1.0000				1.0000		
T3/T4 (33.8%)	**<0.0001**	4.5180	3.0690	6.6510	0.4445	0.7727	0.3989	1.4966
**Metastasis**								
M0 (88.7%)		1.0000				1.0000		
M1 (11.3%)	**<0.0001**	12.0300	8.0760	17.9100	**<0.0001**	5.2895	3.2799	8.5303
**Pathological stage**								
I/II (63.2%)		1.0000				1.0000		
III/IV (36.8%)	**<0.0001**	6.8300	4.4650	10.4500	**0.0006**	4.0809	1.8324	9.0887
**Histological grade**								
Grade 1/2 (46.9%)		1.0000				1.0000		
Grade 3/4 (53.1%)	**<0.0001**	3.3350	2.1480	5.1760	**0.0418**	1.6327	1.0183	2.6177
**ACE2**	**<0.0001**	0.8075	0.7485	0.8712	**<0.0001**	0.8023	0.7375	0.8729

## Abbreviations

ACE2: angiotensin-converting enzyme 2
ccRCC: clear cell renal cell carcinoma
COVID-19: coronavirus disease 2019
SARS-CoV-2: severe acute respiratory syndrome coronavirus 2
OS: overall survival
RFS: relapse-free survival
TCGA-KIRC: The Cancer Genome Atlas-Kidney Renal Clear Cell Carcinoma
GEO: Gene Expression Omnibus
TIP: Tracking Tumor Immunophenotype
TAM: tumor associated microenvironment
CTLs: CD8+ cytotoxic T lymphocytes
