# Supplementary material for: ACE2 Correlated With Immune Infiltration Serves As A Novel Prognostic Biomarker In Clear Cell Renal Cell Carcinoma: Implication For COVID-19
Source: Int J Biol Sci. 2021 Jan 1;17(1):20–31. doi: 10.7150/ijbs.51969 (PMC7757050; doi:10.7150/ijbs.51969)

Supplementary Figure 1. The prognostic roles of CD8 Memory, CD8 Effector, Th cell, Monocytes CD16, pDC, NK and Plasma infiltration in ccRCC.

Supplementary Figure 1

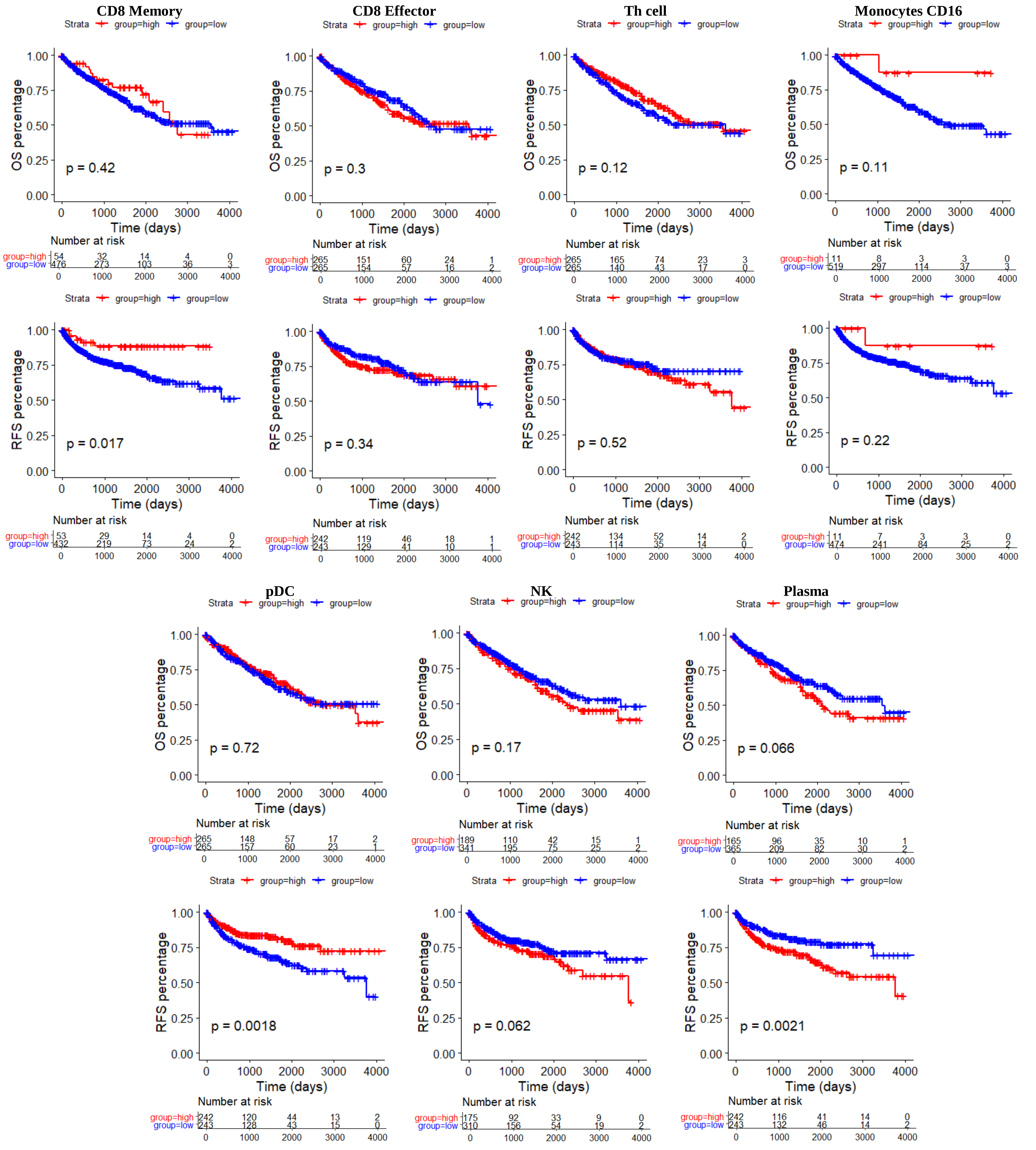

Supplement: Supplementary file 1 — Supplementary figure. [file ijbsv17p0020s1.pdf]
